# Effects of sodium citrate on the structure and microbial community composition of an early-stage multispecies biofilm model

**DOI:** 10.1038/s41598-020-73731-8

**Published:** 2020-10-06

**Authors:** Yuan Yao, Yang Pu, Wing Yui Ngan, Karin Kan, Jie Pan, Meng Li, Olivier Habimana

**Affiliations:** 1 School of Biological Sciences, The University of Hong Kong, Pokfulam, Hong Kong Special Administrative Region China; 2 Institute for Advanced Study, Shenzhen University, Shenzhen, China; 3 The University Hong Kong Shenzhen Institute of Research and Innovation (HKU-SIRI), Shenzhen, Guangdong Province China

**Keywords:** Applied microbiology, Biofilms, Microbial communities, Environmental microbiology

## Abstract

In recent years, most biofilm studies have focused on fundamental investigations using multispecies biofilm models developed preferentially in simulated naturally occurring low-nutrient medium than in artificial nutrient-rich medium. Because biofilm development under low-nutrient growth media is slow, natural media are often supplemented with an additional carbon source to increase the rate of biofilm formation. However, there are knowledge gaps in interpreting the effects of such supplementation on the resulting biofilm in terms of structure and microbial community composition. We investigated the effects of supplementation of a simulated freshwater medium with sodium citrate on the resulting structure, bacterial community composition, and microbial network interactions of an early-stage multispecies biofilm model. Qualitative and quantitative analyses of acquired confocal laser scanning microscopy data confirmed that sodium citrate supplementation distinctly increased biofilm biomass. Sequencing data revealed that the microbial community structure of biofilms grown in sodium citrate-supplemented conditions was characterized with increased relative abundance and dominance of Proteobacteria compared with that of biofilms grown in sodium citrate-free conditions. Our findings suggest that the supplementation of a low-nutrient medium with a carbon source in experiments involving multispecies biofilms may lead to structural and compositional biases of the microbial community, causing changes in biofilm phenotype.

## Introduction

Although the use of in vitro monospecies biofilms and nutrient-rich media has been widely applied in the context of fundamental investigations, realistic multispecies biofilm models have gained much attention in recent years. Given that the microbial systems in natural and engineered environments usually comprise consortia of highly diverse microbial communities organized in the form of biofilms, investigations focusing on multispecies biofilms has emerged as a relevant topic for elucidating the behavior of targeted microorganisms within complex systems^1,2^.

Compared to monospecies biofilm development, the establishment of multispecies biofilm model is a challenging feat, considering the existing complex biological processes linked to their high bacterial diversity^3^ where various forms of interspecies interactions are involved^4^. Therefore, simulating multispecies biofilm within a laboratory setting has always been considered an arduous task, which explains why most published studies have opted to investigating monospecies biofilm models or, in the best case, biofilm models involving two to three different species. Nevertheless, one of the caveat of performing in vitro experiments is associated with the choice of growth medium, regardless of which type of monospecies or multispecies biofilm model is being investigated. In situations where the composition of the inoculum is known, an appropriate medium can be selected for optimal biofilm growth. For monospecies biofilms, past studies have shown that biofilm development outcomes are significantly affected by the selected growth medium^5,6^. In oral biofilm related research, where both monoculture and multispecies biofilm models are typically employed, growth media as well as specific nutrients were also shown to affect growth rate and biofilm composition outcomes^7–12^. More specifically, ecological plaque hypothesis (a hypothesis to explain the relationship between the dental plaque microflora and the host in health and disease, and to identify new strategies for disease prevention) proposed by Marsh assumed that the discrepancy in the microbial composition of oral biofilms impacted by both environmental factors and nutrient availability was found to contribute to the enrichment of disease-associated pathogens^13^. However, in experiments in which biofilm models constituting a plethora of microorganisms need to be investigated, the growth medium is generally obtained from a nonsterile natural or simulated source^14,15^.

In experiments involving the use of nonsterile medium, the issue of repeatability or inconsistent biofilm outcomes may arise due to the dynamic chemical conditions of the natural source and the high diversity of initial microorganisms. For instance, the nutrient contents in freshwater environments can be affected by random factors such as weather alteration, seasonal shifts, or anthropogenic activities^16,17^. Moreover, the speed of biofilm formation may be low in freshwater environments because unlike nutrient-rich media, nonsterile natural media usually contain lower levels of essential nutrients. For example, in nutrient-rich medium, biofilm formation generally occurs within one week, whereas in the natural river medium, at least 15 days are required for the formation of a thin patchy biofilm^18^.

One strategy commonly used to accelerate biofilm formation in nutrient-poor media is the addition of an organic carbon source. In biofilm experiments using nutrient poor media, sodium citrate or sodium acetate is occasionally supplemented as the sole carbon source for accelerating biofilm development in monospecies biofilm models^19–22^ or multispecies consortia^23–26^. It is through carbon source supplementation that such studies were able to accelerate biofilm development processes needed for conducting timely quantitative and qualitative biofilm analyses, thereby providing an attractive means for experimental productivity.

Though the addition of substrates, such as sodium citrate, is an ideal way to increase the rate of biofouling, biofilm formation, and biomass level in monospecies models, it is unclear whether such addition is similarly beneficial in multispecies models. In multispecies biofilm models, we hypothesized that the addition of sodium citrate may alter the multispecies community composition as well as its microbial succession. While sodium citrate is likely to accelerate biofilm development in multispecies biofilms, how substrate addition may affect experimental outcomes in terms of biofilm structure and microbial community composition remains unclear, thereby causing potential experimental biases.

There is currently a plethora of various in vitro biofilm models used in the context of environmental or health-related research, however, the selection of either dynamic- or static-forms of systems will ultimately depend on the original conditions the selected model was meant to simulate. While, static biofilm harvesting systems are perhaps the most widely used in research due to their simplicity and other positive attributes^12,27,28^, they are not ideal in research experiments meant to simulate freshwater environments characterized by continual flow and shear conditions, as well as constant renewal of nutrients. In the present study, we sought to investigate the effects of sodium citrate supplementation on a previously described multispecies biofilm models, using a dynamic simulated irrigation water distribution system^29^. The structure and microbial community composition of biofilms in the absence and presence of sodium citrate were compared at different time points of their early-stage formation.

## Materials and methods

### Multispecies biofilm models

#### Setup of freshwater biofilm models

**Figure 1:**
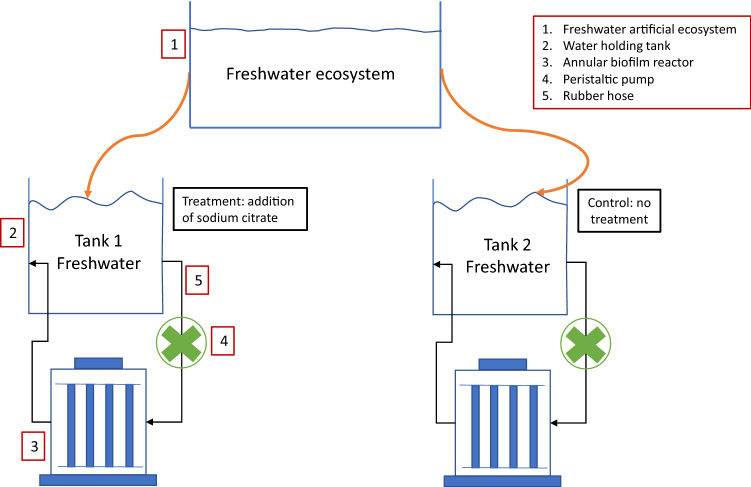
Schematic diagram of the experimental setup used for generating freshwater biofilm models.

The setup of a standardized simulated freshwater source and biofilm model has previously been described in detail^29^ and is schematized in Fig. 1. Briefly, freshwater from a controlled and standardized artificial ecosystem was drawn and directed into two holding tanks 1 and 2. The physicochemical profile and baseline of the artificial freshwater source were monitored throughout the study. Tank 1 functioned as the treatment tank to which sodium citrate was systematically added during biofilm experiment, whereas tank 2 served as the control for freshwater stemming from a healthy ecosystem. Each holding tank was first filled with 5-L aquarium water and connected to an individual Annular Biofilm Reactor (ABR, BioSurface Technologies Corp., Bozeman, MT, US) of 1-L working volume and operated in the recirculation mode. Each ABR could hold up to 40 coupon surfaces made of stainless steel on which biofilms could be harvested for systematic analysis.

#### Study design

For each biofilm system, the freshwater in the holding tank was replaced twice a week as previously described^29^ to ensure a continuous nutrient supply for biofilm growth in each reactor. In the treatment group, sodium citrate was added to the holding tank at a final concentration of 1 mM immediately following every water replacement event. Biofilms were collected for confocal laser scanning microscopy (CLSM) observation and quantification, as well as genomic DNA extraction, at four time points: days 7, 14, 21, and 28, to investigate the shifts in biofilm structure and microbial succession during the early stages of biofilm formation. Throughout the study, biofilms of less than a month were defined as early-stage biofilms, based on a similar definition proposed by Ngan and Habimana^29^. The extracted DNA were sent to Novogene for 16S rRNA gene pyrosequencing and network analysis. All experiments at selected time points under both control and treatment conditions were repeated three times.

### Qualitative and quantitative assessment of biofilms

Biofilm structure was qualitatively assessed from the acquired CSLM (Zeiss LSM 780, Jena, Germany) data. All samples were first stained with 5 mM Syto 9 Green Fluorescent Nucleic Acid Stain (Catalog number: S34854, Thermofisher, Waltham, Massachusetts, United State) without affecting the original biofilm structure. Stained samples were then incubated in a dark box for 30 min. Subsequently, 3D image stacks of random areas of the biofilm samples were created. For biofilm grown in the absence of sodium citrate, an LD Plan-Neofluar 40x/0.6 Korr Ph2 M27 objective lens was used in the acquisition of biofilm stack, via confocal laser scanning microcopy, achieving × 40 magnification. For biofilms supplemented with sodium citrate, an EC Plan-Neofluar 20x/0.50 Ph2 M27 objective was used during CLSM stack acquisitions, achieving × 20 magnification. The rational for using different objectives can be explained by the level of biomass generated by the supplementation of sodium citrate, or lack thereof. We found that the × 40 objective was ideal for acquiring control biofilms stacks, however, the same objective used on biofilm supplemented with sodium citate led to loosing intricate details of the biofilm’s structural properties, which were best observed using a 20 × objective. Three to six 3D stacks were taken for each sample, depending on the sample size, to assure sufficient data for subsequent quantitative analyses.

Qualitative assessments of the biofilms were performed by processing the acquired microscopy data using Fiji software tools (developed by contributors worldwide; the website is hosted by LOCI at the University of Wisconsin-Madison). The quantitative analysis of the microscopy data was performed using the image analysis tool PHLIP running in MATLAB developed by Dr. J. Xavier (https://phlip.sourceforge.net/phlip-ml). The following quantitative parameters were evaluated: total biovolume (µm^3^), surface coverage (µm^2^), mean thickness (µm), and biofilm roughness (dimensionless)^30^. SPSS (IBM SPSS Statistics 24.0) was used to performed an analysis of variance for comparing biofilm groups acquired at different time points.

### DNA extraction, sequencing, and metagenomic analysis

Genomic DNA (gDNA) of biofilms grown under different conditions and sampled at selected time points were extracted using Quick-DNA Fecal/Soil Microbe Kits (Catalog No. D6010, Zymo Research, Irvine, CA, USA) as previously described^29^.

All gDNA samples were sent to Novogene (Novogene Bioinformatics Technology, Beijing) for amplicon V3-V4 bacterial sequencing and analyses (Forward primer: 3′-CCTAYGGGRBGCASCAG-5′ , reverse primer: 3′-GGACTACNNGGGTATCTAAT-5′ ). During the transfer, all samples were kept in an isolation box with dry ice to prevent thawing and degradation. An amplicon library with fusion primers (16S V3-V4) was first constructed with dual index and adapters for PCR. Phusion High-Fidelity PCR Master Mix (New England Biolabs, Ipswich, MA, USA) was used for all PCR reactions. As a standard procedure at Novogene (Novogene Bioinformatics Technology, Beijing), PCR products underwent in-house quantification and purification procedures. A TruSeq DNA PCR-free sample preparation kit (Illumina, San Diego, CA, USA) was used following the manufacturer’s instruction for generating sequencing libraries, which were then assessed using the Qubit 2.0 Fluorometer (Thermo Scientific) and finally sequenced on an Illumina HiSeq2500 platform, generating 250-bp paired-end reads.

The obtained paired-end reads were merged using FLASH^31^ following the removal of barcodes and primer sequences. Quality filtering was performed on raw reads using specific filtering conditions to obtain high-quality clean reads^32^ according to the QIIME quality control process^33^. The obtained effective tags were compared with a reference database (Gold database) using the UCHIME algorithm^34^ to remove chimeric sequences. Sequence analysis was performed using the UPARSE algorithm^35^, where sequences with a similarity index of 97% or above were assigned to the same operational taxonomic units (OTUs). Screening for species annotation to obtain a representative sequence for each OTU was performed using the GreenGene Database^36^ based on RDP Classifier^37^. The phylogenetic relationships of different OTUs, differences between dominant species in samples (groups), and multiple sequence alignments were analyzed using PyNAST v1.2^38^ against the “Core Set” dataset in the GreenGene database.

Statistical methods including t-test, MetaStat (calculated using R), LEfSe (calculated by LEfSe software), Anosim (performed using R), and MRPP (performed using R) were used to assess the significance of differences in the structure and microbial community composition between the groups. The workflow of data analysis is provided in S1.

### Network analysis

Network analysis of features and taxa in each sample was performed using CoNet app 1.1.1 beta^39^ in Cytoscape 3.6.0 at the following settings: Pearson correlation, Spearman correlation, Bray–Curtis dissimilarity, and Kullback–Leibler dissimilarity were calculated, and the correlation threshold was 0.3. The initial edge selection was set to include the top 100 and bottom 100 scoring edges. Edges supported by less than two metrics were removed. The significance of edges was calculated and renormalized based on 1000 bootstrap iterations, and *p* values were adjusted using the Benjamini–Hochberg correction for multiple tests. Only edges with *p* ≤ 0.05 were included in the network.

## Results

### Qualitative and quantitative assessment of freshwater biofilm models grown in the presence vs absence of sodium citrate

**Figure 2:**
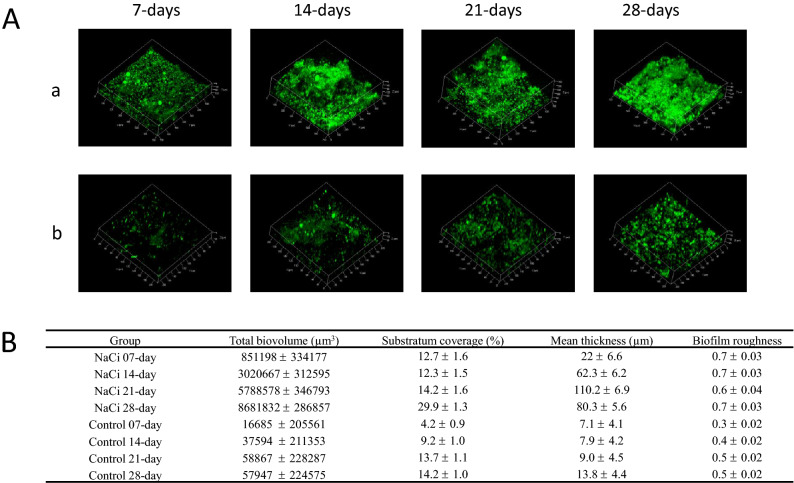
Qualitative (**A**) and quantitative (**B**) assessments of freshwater biofilms grown under control and sodium citrate conditions over time. (**A**) 3D reconstructed projections of CLSM-acquired stacked images of Syto 9-stained biofilms grown under a) 1 mM sodium citrate-supplemented conditions (NaCi) and b) sodium citrate-free conditions (Control). (**B**) The quantitative analysis of 3D biofilm images by PHLIP describing the total biovolume (µm^3^), substratum coverage (µm^2^), mean thickness (µm), and biofilm roughness (dimensionless). Error represents the standard deviation of the mean of at least *n* = 3 fields of view from three independent experiments for each time point, days 7, 14, 21, and 28.

To determine the effect of sodium citrate supplementation on fresh water biofilm structure over a four-week period, biofilms were harvested using two ABR systems run in parallel, one under the presence and another under the absence of sodium citrate (NaCi and control). The results of the qualitative and quantitative analyses of the acquired microscopy data are presented in Fig. 2.

The visualization of 3D reconstructed biofilms (Fig. 2A) revealed distinct outcomes between the sodium citrate-supplemented and sodium citrate-free (control) conditions. Biofilms grown in the presence of sodium citrate were characterized by heterogeneous structures from day 7 to day 28 of the experiment and exhibited completely developed 3D structured masses from day 14. In contrast, Biofilms grown in the absence of sodium citrate were characterized as homogenously spread microcolonies and aggregates that gradually colonized the surface from day 7 to day 28 of the experiment. Quantitative analysis of the microscopy data (Fig. 2A) validated the qualitative observations. Under sodium citrate conditions, the total biovolume was 50 times (*p* < 0.01) and 100 times (*p* < 0.01) higher on day 7 and 28 than that on the corresponding days under control conditions, which is 1.6 × 10^4^, and 5.7 × 10^4^ µm^3^, respectively. Under sodium citrate conditions, surface coverage remained in the range of 12.7 to 14.2 µm^2^ from day 7 to day 21 before doubling to 29.9 µm^2^ on day 28. Under control conditions, surface coverage doubled from day 7 to day 14 and gradually increased thereafter. Compared with surface coverage under control conditions, that under sodium citrate conditions was three times and two times higher on day 7 (*p* < 0.01) and day 28 (*p* < 0.001), respectively. Biofilm thickness quadrupled from 22 µm on day 7 to 80.3 µm on day 28 under sodium citrate conditions, whereas doubled from 7.1 µm on day 7 to 13.8 µm on day 28 under control conditions. Compared with biofilm thickness under control conditions, that in sodium citrate conditions was significantly higher between days 14 and 28 (*p* < 0.001). Biofilms grown under sodium citrate conditions exhibited higher average roughness value of 0.7 (*p* < 0.001) during day 7 to day 28. In contrast, biofilms grown under control conditions exhibited very low roughness values ranging from 0.3 on day 7 to 0.5 on day 28. The raw values of quantitative analysis of each biofilm repeat were plotted as box plots (Supplementary figure 1).

### Microbial community profile of freshwater biofilm models grown in the presence vs absence of sodium citrate

**Figure 3:**
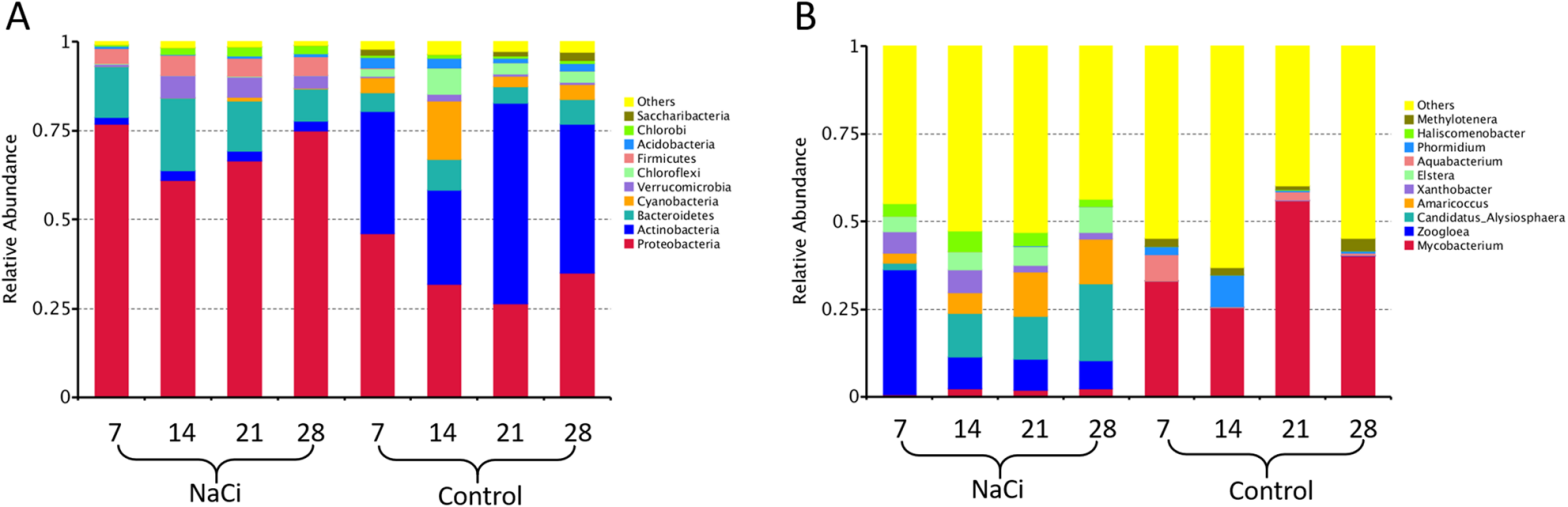
Relative abundance of the top 10 most abundant microbes for 16srRNA sequnces across different treatments and time points. Data are average of three repeats. Group names on X-axis “A1, A2, A3, A4” represent week 1 (7-days), week 2 (14-days), week 3 (21-days), week 4 (28-days) biofilms incubated under sodium citrate supplementation, respectively. Group name on X-axis “B1, B2, B3, B4” represent week 1 (7-days), week 2 (14-days), week 3 (21-days), week 4 (28-days) biofilms incubated under control condition. (**A**) Histogram of relative abundance at the phyum level. (**B**) Histogram of relative abundance at the genus level.

The top 10 taxa in different taxonomic ranks were selected to generate distribution histograms of relative abundance. The distribution at the phylum (Fig. 3A) and genus (Fig. 3B) levels are presented in Fig. 3 for biofilms grown in the presence and absence of sodium citrate (NaCi and Control, respectively). The three most abundant prokaryotic phyla (Fig. 3A) were Proteobacteria (70.0%), Bacteroidetes (14.4%), and Firmicutes (5.0%) in biofilms grown under sodium citrate conditions, where Actinobacteria (40.0%), Proteobacteria (35.0%), and Cyanobacteria (7.1%) in those grown under control conditions. The three most abundant genera (Fig. 3B) were *Zoogloea (15.4%), Candidatus_Alysiosphaera (12.2%), and Amaricoccus* (8.5%) in biofilms grown under sodium citrate conditions, where *Mycobacterium* (38.7%), *Phormidium* (3.1%), and *Aquabacterium* (2.7%) in those grown under control conditions. Changes in the microbial community composition were also observed over the course of the experiment. In biofilms grown under sodium citrate conditions, the relative abundance of the most dominant phylum Proteobacteria decreased from 76.9% on day 7 to 61% on day 14 and then gradually increased to 66.4% on day 21 and 75.0% on day 28 (Fig. 3A). Under control conditions, the relative abundance of the most dominant phylum Actinobacteria increased from 26.6% on day 14 to 56.4% on day 21 (Fig. 3A). Further, under sodium citrate conditions, the relative abundance of the most dominant genus *Zoogloea* decreased from 35.6% on day 7 to 9% on day 14 and remained stable thereafter until day 28 (Fig. 3B). Under control conditions, the relative abundance of the most dominant genus *Mycobacterium* decreased from 56.0% on day 21 to 25.6% on day 14 (Fig. 3B). The microbial composition of individual biofilms at phylum level and genus level were presented in Supplementary figure 2 and 3, respectively.

### Biofilm community structure analysis of biofilms grown in the presence vs absence of sodium citrate

**Figure 4:**
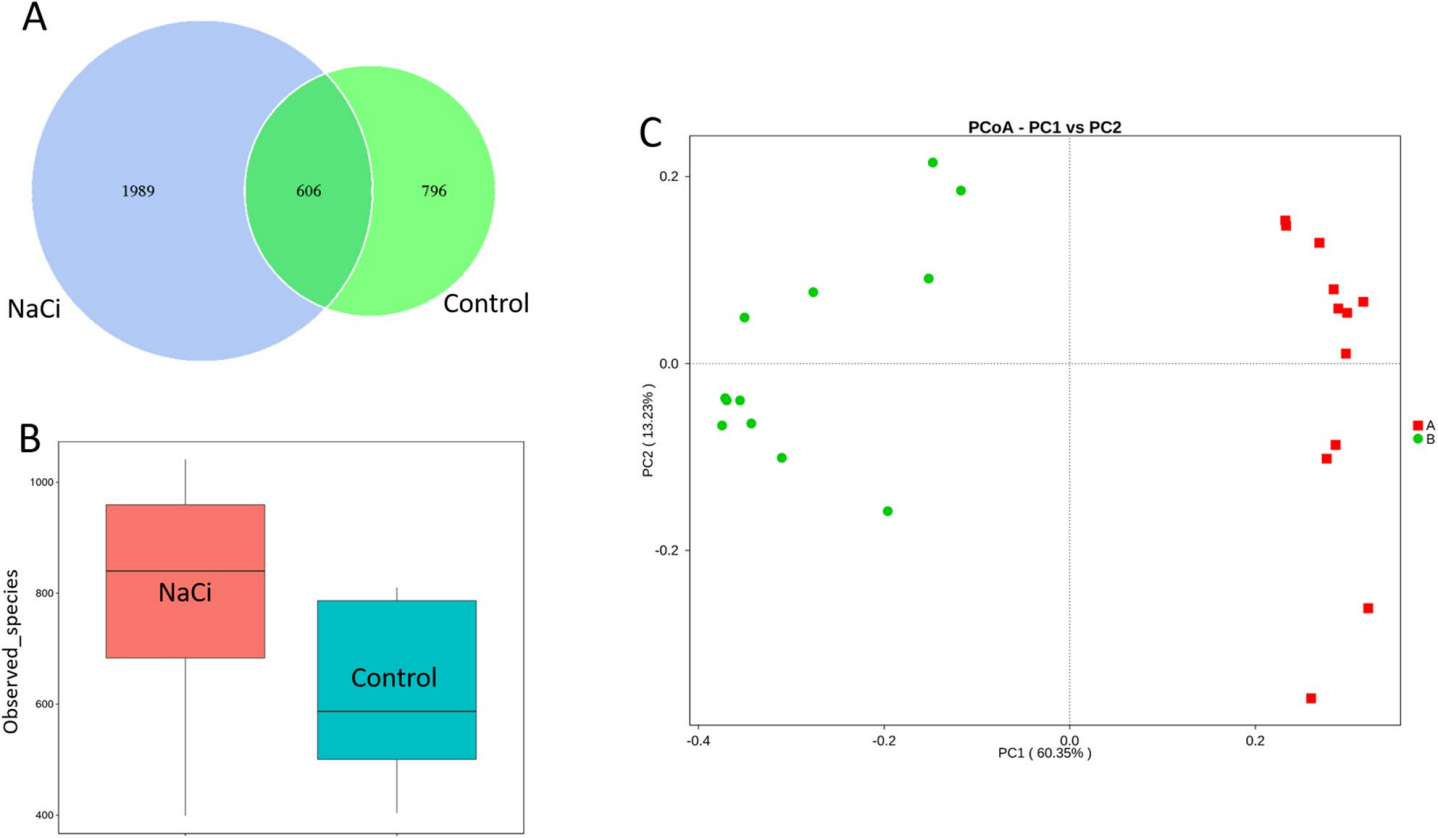
Microbial community structure analysis of biofilms grown in control vs sodium citrate-supplemented conditions. (**A**) Venn diagram displaying the number of shared and unique OTUs of modeled biofilms. (**B**). Boxplot diagram of alpha-diversity results of modeled biofilms. Wilcoxon rank-sum test, *p* < 0.05 (**C**). Principal coordinate analysis based on weighted UniFrac distance for biofilms grown under sodium citrate-supplemented (red dots) and control (green dots) conditions.

The microbial community structures were further analyzed using 16S rRNA gene sequencing data to compare the growth between sodium citrate-supplemented and sodium citrate-free conditions. Venn diagram (Fig. 4A) revealed that freshwater biofilms grown under sodium citrate conditions had a more diverse community with 1989 observed species compared with 796 species observed in biofilms grown under control conditions. Alpha-diversity analysis suggested that biofilm grown under sodium citrate conditions presented a significant higher species diversity than that found in control conditions. (Wilcoxon rank-sum test, *p* < 0.05) (Fig. 4B).

Additionally, a principal coordinate analysis was performed to evaluate the separation patterns of the biofilm community structures between the control and NaCi conditions (Fig. 4C). Based on phylogenetically weighted UniFrac distance, all samples of biofilms grown under sodium citrate conditions were clearly clustered into one group along the first principal coordinate and separated from those of biofilms grown under control conditions. The separation pattern was consistent with the α-diversity results, which showed a clear separation of community structures in the tested groups between the sodium citrate-supplemented and sodium citrate-free conditions.

### Microbial interaction network analysis of co-occurrence and co-exclusion patterns

**Figure 5:**
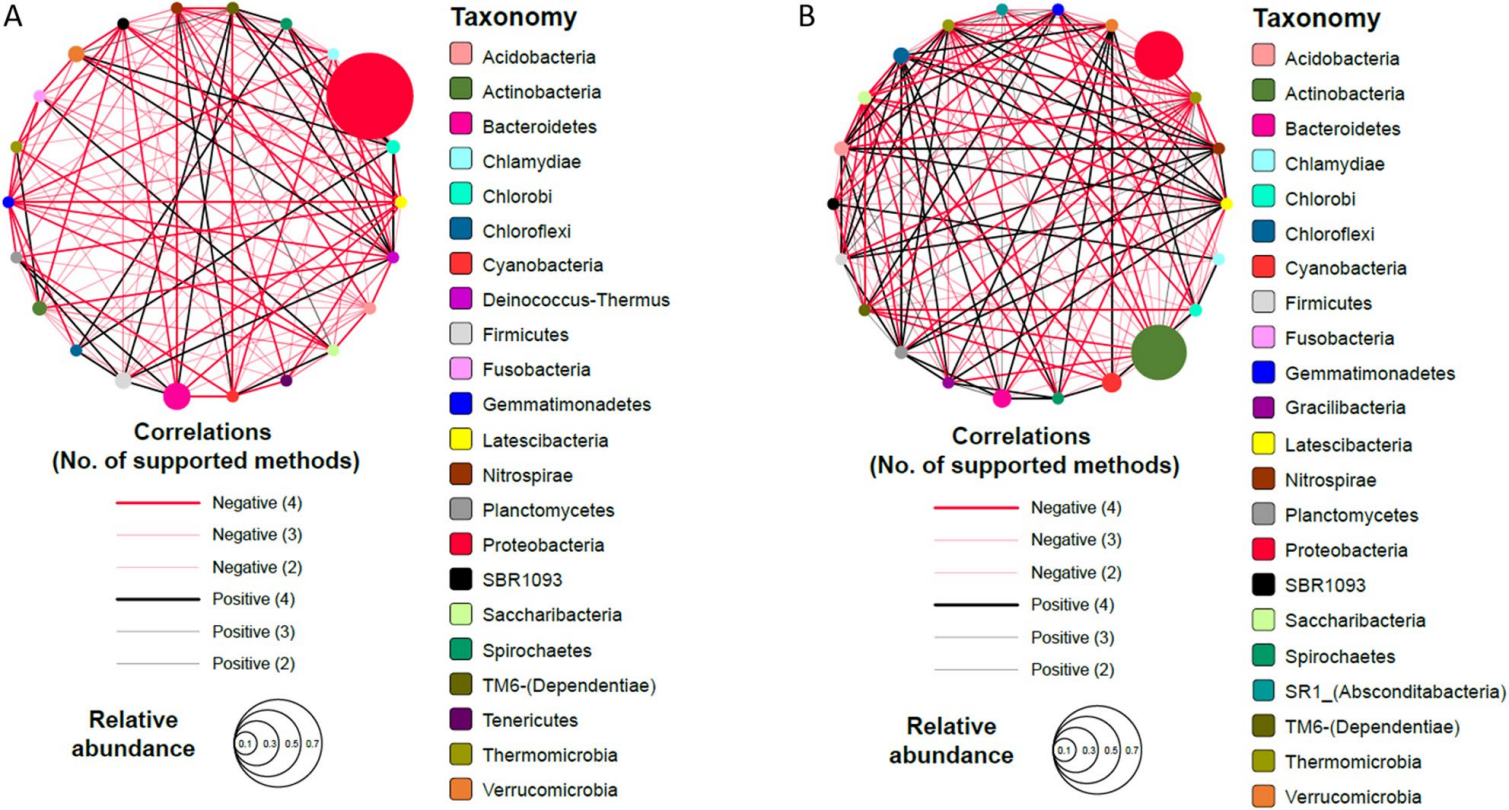
Correlation networks of microbial communities at the phylum level in biofilms grown under sodium citrate-supplemented (**A**) and control (**B**) conditions. Each node represents one bacterial phylum. The increasing sizes of the nodes indicate increasing relative abundances. The nodes are connected by edges. The edge font is proportional to the number of supported methods showing significant correlations. The numbers on the edges represent the correlation coefficients. The edges in black and red indicate positive (co-occurrence) and negative (co-exclusion) correlations, respectively.

The correlation analysis of microbial communities was performed at the phylum level, as shown in Fig. 5. In this figure, the node size is proportional to the relative abundance of each phylum. The edge font is proportional to the number of supported methods that presented significant correlation (*p* < 0.05). Black and red edges represent positive and negative correlations, respectively. Top 100 and bottom 100 scoring edges were selected. This revealed 22 phyla in each group, exhibiting co-inclusion or co-exclusion with other phyla; of these, 19 phyla were common and three were distinct between sodium citrate conditions (A) and control conditions (B). Compared with biofilms in sodium citrate conditions (average degree = 12), those in control conditions showed a more complex network interconnection with an average of 13.8 (average degree) connections per node. In addition, the network of control conditions showed higher composition of positive correlations (38.2%) compared with the network of sodium citrate conditions (17.4%).

In the interaction network of biofilms grown under sodium citrate conditions, Proteobacteria represented the largest node (indicating that it was the most dominant phylum) with the highest number of co-exclusion interactions (negative degree = 15). In contrast, in the interaction network of biofilms grown under control conditions, Actinobacteria represented the largest node with the highest number of co-exclusion interactions (negative degree = 18), and Proteobacteria represented the second largest node with 11 co-exclusion interactions. Furthermore, the highest number of co-inclusion interactions was shown by the TM6 (Dependentiae) phylum (positive degree = 6) in the interaction network of biofilms grown under sodium citrate conditions and by the Chloroflexi phylum (positive degree = 10) in that of biofilms grown under control conditions.

## Discussion

Sodium citrate and sodium acetate are the most commonly used carbon sources for supplementing natural or artificial low-nutrient media to accelerate the formation of monospecies or multispecies biofilm in laboratory-based biofilm experiments^19–26^. However, the potential biased effects caused by supplementation with such carbon sources on the structural attributes, microbial community composition, and microbial interaction networks of biofilms remain unexplored. To close this knowledge gap, we investigated the effects of a commonly used carbon source, sodium citrate, on the structure, microbial community composition, and microbial interactions of an early-stage multispecies biofilm model previously described in the context of simulated irrigation water distribution systems^29^. The results of our analyses revealed that the addition of sodium citrate creates a biased biofilm model in terms of an inconsistent microbial composition that may affect the outcome of the resulting biofilm.

With respect to biofilm biomass accumulation, as expected the supplementation of a nutrient-poor artificial medium with sodium citrate successfully increased the rate of biofilm formation, as indicated by the enhanced 3D biofilm structure. Notably, the total biovolume, mean thickness, and roughness parameters increased significantly in biofilms grown in sodium citrate-supplemented medium. This finding is in accordance with findings in previous studies in which supplementary carbon source could increase microbial biomass^40,41^. Bester et al.^40^ revealed that compared to carbon-limited conditions, carbon-replete environments led to 40% increase in planktonic cell yields of *Pseudomonas* sp*.* biofilms. Another study showed that carbon sources such as glucose, sucrose and acetate presented a significant enhancement in microalgae growth^41^.

In particular, comparison of the microbial community profiles of biofilms between sodium citrate-supplemented and sodium citrate-free conditions revealed that the sodium citrate supplementation resulted in experimental bias in the resulting biofilm phenotypes. Sodium citrate supplementation increased the abundance of the Proteobacteria phylum (70.0%) such that it became the most dominant phylum under sodium citrate conditions. This is a phylum of gram-negative bacteria that includes a wide variety of pathogenic genera, such as *Salmonella*, *Vibrio*, *Helicobacter*, *Yersinia*, *Legionellales*, and *Pseudomonas*^42^. Such a shift in the microbial community structure may be attributable to the fact that some genera under Proteobacteria favor citrate as their sole carbon source such as *Salmonella*^43^. This shift also caused significant changes in the microbial network dynamics in terms of increased co-exclusion interactions of Proteobacteria with other phyla, suggesting the potential nutritional advantage to Proteobacteria. Previous studies have described biofilms primarily composed of Proteobacteria, could influence the overall structural attributes, due to their distinct extracellular polymeric substances (EPS) and biofilm viscoelastic properties^44^. In this study, the thickness and roughness of biofilms grown under sodium citrate conditions varied toward the end of the experiment on day 21 and 28, which could be explained by the potential sloughing of the biofilms in the reactor. Sloughing have been demonstrated as one of the distinct models of biofilm dispersal, for biofilm suddenly sheds a portion of its structure especially at a later stage^45–47^. However, whether the sloughing was attributable to both the significant biofilm thickness and shear conditions within the reactor or to the intrinsic viscoelastic property of the biofilms allowing for effortless sloughing still remains unclear and should be investigated in the future.

In sharp contrast, biofilms grown under sodium citrate-free conditions showed less susceptibility to sloughing under experimental shear conditions, indicating that the high abundance of Actinobacteria may have increased the cohesiveness of the biofilm. Previous investigations on Actinobacteria biofilms have revealed that these biofilms are highly adherent and may promote the recruitment of other cells to its structure. In one recent study, *Micrococcus luteus*, a species of the Actinobacteria phylum, was found to enhance the adhesion of other bacteria to the biofilm^48^. Two other species of the Actinobacteria phylum, *Corynebacterium renale* and *C. pilosum*, are also associated with increased adherence to various bacteria^49^.

Future studies are clearly warranted to further elucidate the impact of microbial community structure on the viscoelastic property of multispecies biofilms. The observed differences in the microbial community profiles caused by sodium citrate supplementation in low-nutrient media may lead to experimental biases in studies focusing on the structural properties of biofilms or specific microbial communities. In the former case, supplementation with sodium citrate or another carbon source to accelerate the turnover of multispecies biofilm models for testing biofilm mitigation strategies could result in the design or engineering of antifouling features that may be inefficient in natural conditions. As an important feature in many types of biofilms including oral, environmental and medical biofilms^50^, the viscoelasticity property of biofilms serves a protective role from external stresses in the form of chemical or mechanical challenges^51^. More specifically, most biofilms in their natural habitats are exposed to external forces applied in a compressive, tensile or shear modes^51^. Given that biofilm EPS are characterized as a multi-component biological material^52–54^, biofilm developments is influenced in a time-dependent manner with series of shifts in elastic and viscous properties. Since biofilm viscoelasticity properties are closely related to biofilm structure and composition, the present study provides a caveat in which researchers should also consider the effects of nutrient supplementation when studying biofilm viscoelasticity and their related response to induced stresses; and by doing so, avoiding potential biased outcomes.

In a microbial ecological context, the beneficial faster turnover of the biofilm model resulting from carbon source supplementation may cause a serious misrepresentation of the microbial community intended for fundamental investigations. In this study, sodium citrate supplementation significantly decreased the relative abundance of Actinobacteria among other phyla. Such biases may hamper accurate fundamental studies on biofilms, especially multispecies biofilm models, in specific simulated environmental conditions.

Although it is understandable that the use of carbon source to accelerate the time-consuming biofilm formation in experimental models is essential, the results of this study suggest the importance of carefully considering the potential biases caused by carbon source supplementation, as they may significantly affect the experimental results. The potential biases in biofilms grown in supplemented media include shifts in the biofilm structure and microbial composition compared with those in biofilms grown in non-supplemented media.

## Conclusion

Our study revealed that adding sodium citrate, a carbon source, to an early-stage multispecies biofilm model significantly affects the biofilm 3D structure, characteristics, bacterial community composition, and network interconnection, thus generating a biased biofilm model for the investigation. The findings provide caveats for investigators intending to accelerate biofilm growth by adding a carbon source in their low-nutrient media for generating multispecies biofilm models.

## Supplementary information

Supplementary Figures.

## Data Availability

Raw sequencing reads have been deposited in the NCBI Sequence Read Archive under the accession number PRJNA594465.
