# Supplementary material for: Effects of sodium citrate on the structure and microbial community composition of an early-stage multispecies biofilm model
Source: Sci Rep. 2020 Oct 6;10:16585. doi: 10.1038/s41598-020-73731-8 (PMC7538881; doi:10.1038/s41598-020-73731-8)
Supplement: Supplementary file 1 — Supplementary Figures. [file 41598_2020_73731_MOESM1_ESM.pdf]

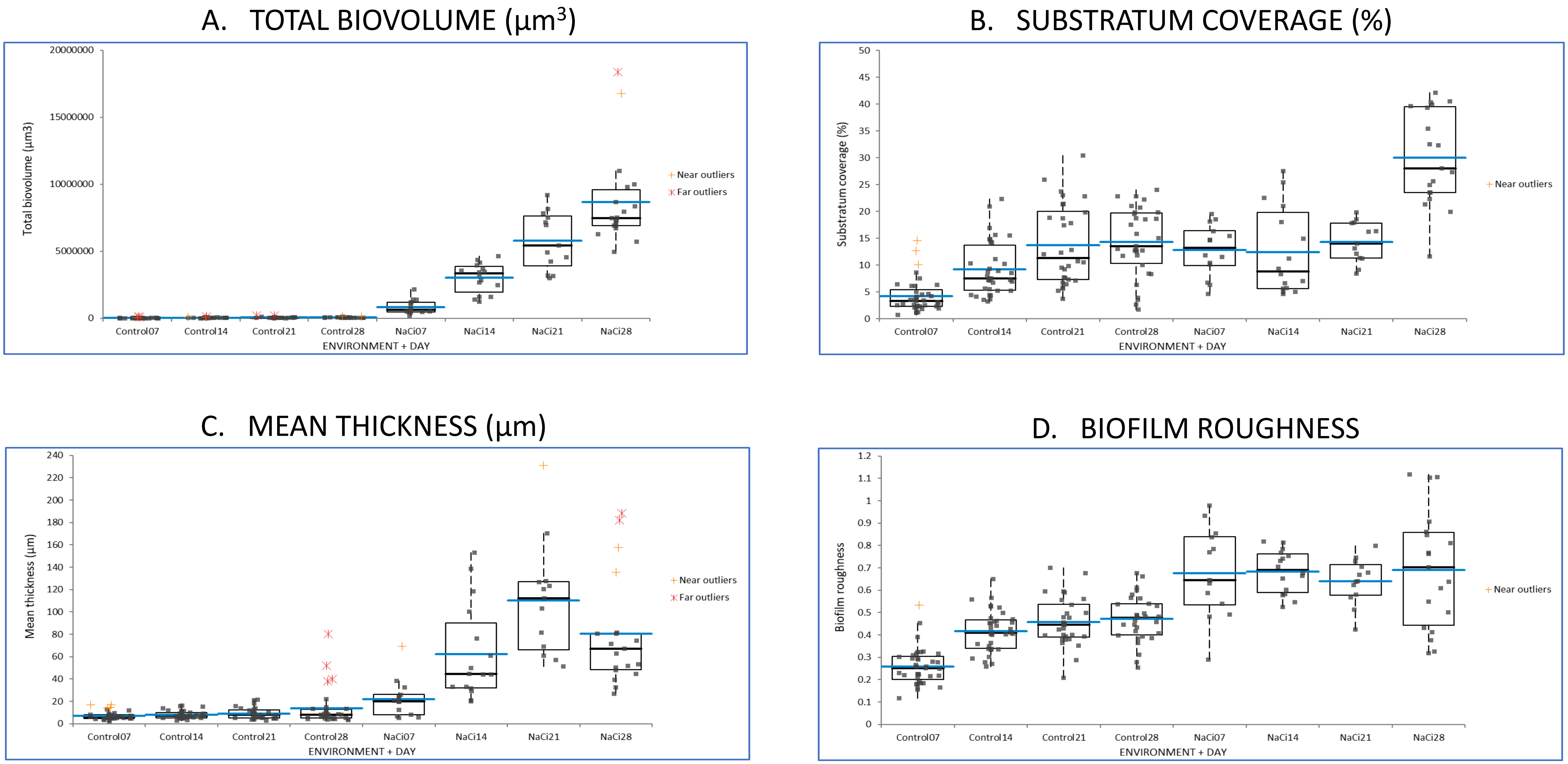

Supplementary figure 1: The quantitative analysis of 3D biofilm images by PHLIP. Raw values of each biofilm repeat were plotted as box plot.

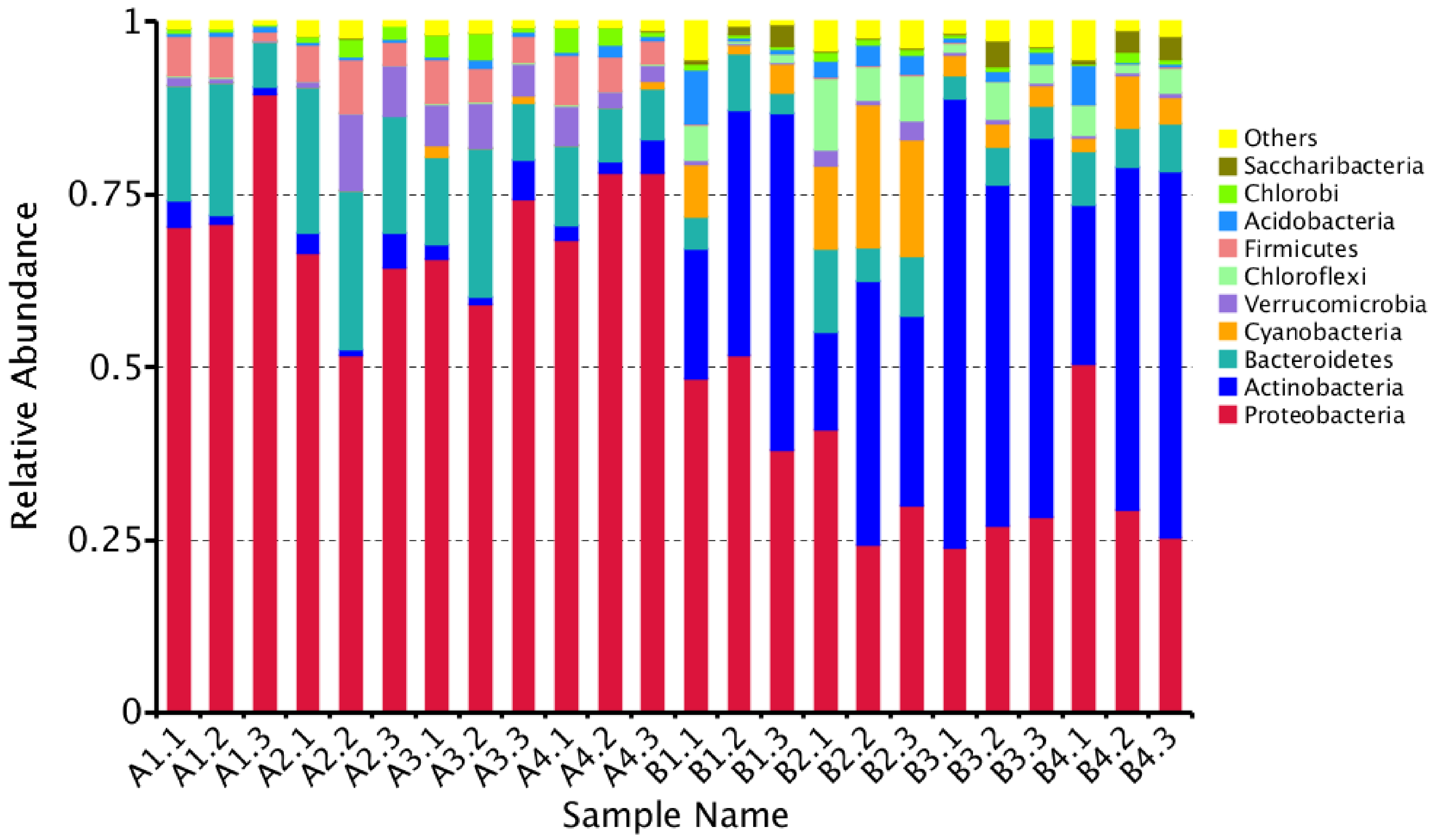

Supplementary figure 2: Relative abundance of the top 10 most abundant phyla for individual biofilms grown in sodium citrate-supplemented and control conditions on days 7, 14, 21, and 28. For group names on x-axis, letter A and B represent biofilm grown in sodium citrate supplemented and control conditions, respectively. The first number following the letter indicates week number, and the second number following the letter indicates repeat number.

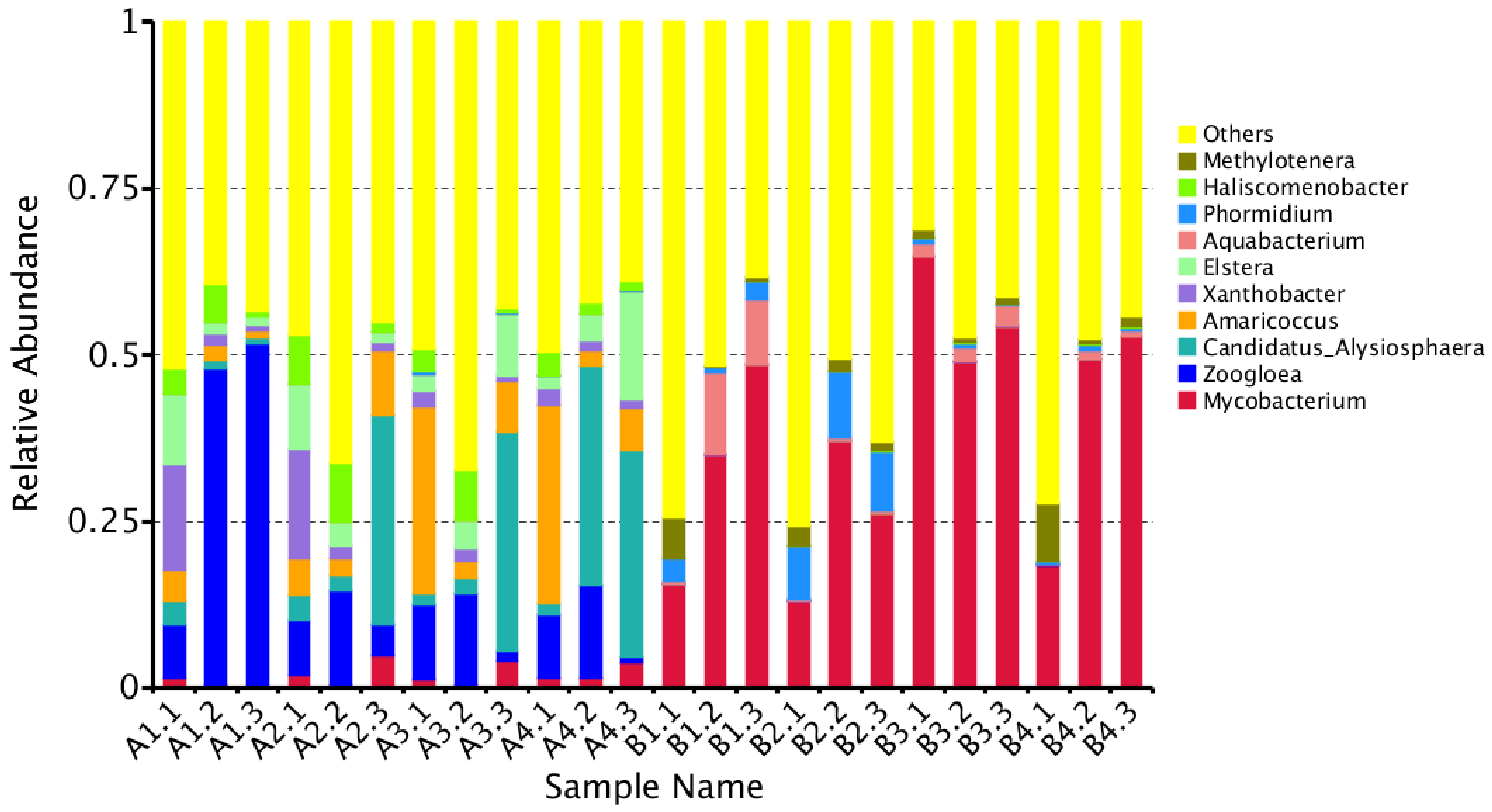

Supplementary figure 3: Relative abundance of the top 10 most abundant genera for individual biofilms grown in sodium citrate-supplemented and control conditions on days 7, 14, 21, and 28. For group names on x-axis, letter A and B represent biofilm grown in sodium citrate supplemented and control conditions, respectively. The first number following the letter indicates week number, and the second number following the letter indicates repeat number.
